# Influenza A(H1N1)pdm09 Virus Suppresses RIG-I Initiated Innate Antiviral Responses in the Human Lung

**DOI:** 10.1371/journal.pone.0049856

**Published:** 2012-11-21

**Authors:** Wenxin Wu, Wei Zhang, J. Leland Booth, Jordan P. Metcalf

**Affiliations:** 1 Pulmonary and Critical Care Division, Department of Medicine, University of Oklahoma Health Sciences Center, Oklahoma City, Oklahoma, United States of America; 2 Department of Microbiology and Immunology, University of Oklahoma Health Sciences Center, Oklahoma City, Oklahoma, United States of America; Centre of Influenza Research, The University of Hong Kong, Hong Kong

## Abstract

Influenza infection is a major cause of morbidity and mortality. Retinoic acid-inducible gene I (RIG-I) is believed to play an important role in the recognition of, and response to, influenza virus and other RNA viruses. Our study focuses on the hypothesis that pandemic H1N1/09 influenza virus alters the influenza-induced proinflammatory response and suppresses host antiviral activity. We first compared the innate response to a clinical isolate of influenza A(H1N1)pdm09 virus, OK/09, a clinical isolate of seasonal H3N2 virus, OK/06, and to a laboratory adapted seasonal H1N1 virus, PR8, using a unique human lung organ culture model. Exposure of human lung tissue to either pandemic or seasonal influenza virus resulted in infection and replication in alveolar epithelial cells. Pandemic virus induces a diminished RIG-I mRNA and antiviral cytokine response than seasonal virus in human lung. The suppression of antiviral response and RIG-I mRNA expression was confirmed at the protein level by ELISA and western blot. We performed a time course of RIG-I and interferon-β (IFN-β) mRNA induction by the two viruses. RIG-I and IFN-β induction by OK/09 was of lower amplitude and shorter duration than that caused by PR8. In contrast, the pandemic virus OK/09 caused similar induction of proinflammatory cytokines, IL-8 and IL-6, at both the transcriptional and translational level as PR8 in human lung. Differential antiviral responses did not appear to be due to a difference in cellular infectivity as immunohistochemistry showed that both viruses infected alveolar macrophages and epithelial cells. These findings show that influenza A(H1N1)pdm09 virus suppresses anti-viral immune responses in infected human lung through inhibition of viral-mediated induction of the pattern recognition receptor, RIG-I, though proinflammatory cytokine induction was unaltered. This immunosuppression of the host antiviral response by pandemic virus may have contributed to the more serious lung infections that occurred in the H1N1 pandemic of 2009.

## Introduction

In 2009, a global outbreak caused by the novel pandemic H1N1 influenza virus spread to numerous countries and infected over 300,000 individuals with at least 16,000 confirmed human deaths worldwide (WHO). The virus was a completely new reassorted virus [1], [2], and the majority of the human population did not have preexisting immunity against it. The influenza A(H1N1)pdm09 virus originated from two swine influenza A virus strains. The new virus has gene segments from a North American H3N2 triple reassortment, classical swine H1N1 lineage, and a Eurasian avian-like swine H1N1 virus. Sequence analysis of this new pandemic virus revealed that hemagglutinin (HA), nucleoprotein (NP), and NS gene segments were derived from the classical swine viruses via the triple reassortant. The PB1, PB2, and PA gene segments were from the North American H3N2 triple reassortment lineage. In addition, the NA and M segments originated from the Eurasian swine virus lineage. The influenza A(H1N1)pdm09 virus is genetically and antigenically distinct from previous seasonal human influenza A H1N1 viruses. Thus, the seasonal influenza vaccines provided little protection [3].

At present, the pathogenesis and transmission of pandemic H1N1/09 influenza virus in humans is still unclear. Animal studies revealed that the pandemic virus replicated better than seasonal H1N1 viruses in the respiratory tracts of the animals, evidenced by enhanced pathogenicity as compared with seasonal influenza viruses in ferrets [4], [5]. Many groups also reported that pandemic H1N1 replicates efficiently in non-human primates, causes more severe pathological lesions in the lungs of infected mice, ferrets and non-human primates than the former circulating human H1N1 viruses [6], [7]. Severity of pneumonia due to pandemic H1N1 influenza virus in ferrets is intermediate between that due to seasonal H1N1 virus and highly pathogenic avian influenza H5N1 virus [8], [9]. One study compared the pathogenesis in mice caused by two different influenza virus subtypes, pandemic H1N1 and H5N1. The results suggest that fatal infections caused by different influenza viruses do not necessarily share the same pathogenesis [10]. Together, these data highlight the need for better understanding the mechanisms underlying the influenza A(H1N1)pdm09 virus in humans.

Epithelial cells are the primary site of viral replication for influenza, although monocytes/macrophages and other leukocytes can also be infected [11]. Influenza virus specific antigen has been found in type 1 and type 2 alveolar epithelial cells, as well as in alveolar macrophages. Viruses initiate infection by binding of the viral HA to sialic acid on the cell surface and enter the cells by receptor-mediated endocytosis. Once inside the cells, influenza virus shuts off host cell protein synthesis and replicates in a fast and efficient way. This process results in host cell apoptosis or death by cytolysis [12]–[14]. However, the host cells respond in several ways to limit viral spreading. The most significant response is production of cytokines and chemokines by epithelial cells and leukocytes via activation of multiple transcriptional and posttranslational systems [15].

Innate immune antiviral responses are the first line of defense against virus infection [16], [17]. Interferons (IFNs) are critical in fighting influenza within host cells [18], [19]. IFNs interfere with viral replication, activate immune cells, such as natural killer cells and macrophages, increase recognition of infection by up-regulating antigen presentation to T lymphocytes, and increase the ability of uninfected host cells to resist new infection by virus. IFN responses to negative-strand RNA viruses, including influenza virus, require the action of Retinoic acid-inducible gene 1 (RIG-I) [20], [21]. This protein resides in the cytoplasm and senses the presence of viral RNA, either double-stranded RNA [22] or single-stranded RNA with a 5′-phosphate [23]. When these RNAs bind RIG-I, a signaling cascade is initiated which culminates in the production of IFNs. The IFNs in turn activate the synthesis of nearly a thousand cellular proteins which have antiviral properties. Sensing of virus presence and cytokine induction via the RIG-I pathway are crucial for successful host defense against infections with RNA viruses [24]. Influenza virus, evolutionally, has developed strategies to prevent host immune activation. In fact, the virulence of some virus strains is due, at least in part, to a deregulation of the innate immune response [25]–[27].

We have developed a human lung organ culture model in order to study the local lung response to human pathogens [28]–[30]. Precision-cut lung slices have frequently been used in toxicology studies and have advantages over the use of isolated, cultured epithelial cells for infectious disease studies. The structural integrity of lung tissue is maintained and this allows for cell-cell interaction in a more complex and native three-dimensional system.

In the present study, we have compared viral replication rates and innate immune responses to influenza A(H1N1)pdm09 virus, seasonal H3N2, and H1N1 strains in our human lung organ culture model. We found that pandemic H1N1 virus suppresses RIG-I induction and has an immunosuppressant effect on antiviral immune responses in infected human lung, which may contribute to the increased lung pathogenicity of this pandemic strain.

## Results

### Both Pandemic and Prototypic Influenza Virus Infect and Replicate in Alveolar Cells of Human Lung

**Figure 1:**
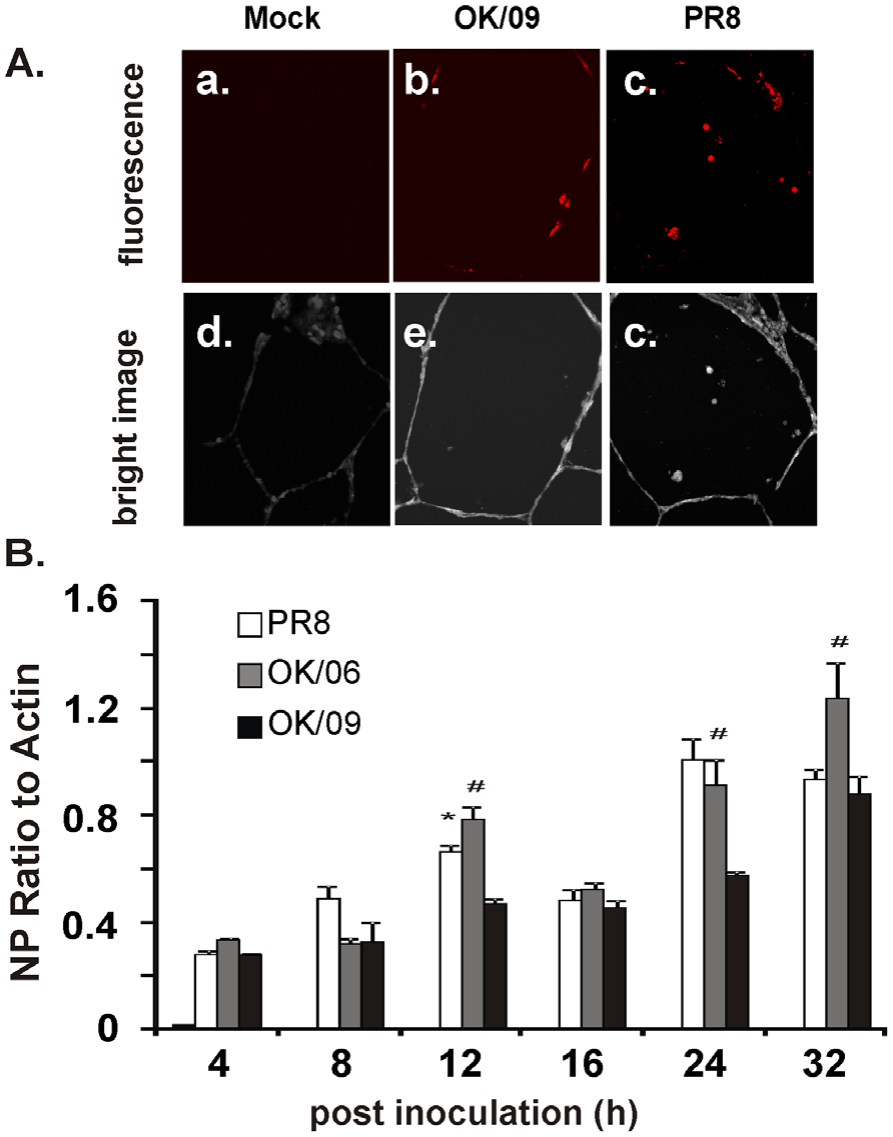
Exposure of human lung to both pandemic and prototypic influenza virus results in viral infection and replication in alveolar cells. (A) The lung slices were processed for immunohistochemistry for detection of viral NP using rabbit polyclonal antibody (red). Panels a, b and c show mock (virus dilution buffer), OK/09 and PR8 infection, respectively. Panels d, e and f are corresponding bright-field images that demonstrate that lung architecture is preserved during the experiment. (B) Replication of influenza virus OK/09, OK/06 and PR8 in the human lung organ culture model. Lung slices exposed to virus at 6×10^6^ PFU/ml were cultured for various times and total cellular mRNA was extracted. Quantitative RT-PCR was performed using Oligo dT as the primer for the first strand synthesis. Primers specific for NP were used to examine NP mRNA expression.

First, we compared infection and replication of influenza A(H1N1)pdm09 virus with a clinical isolate of seasonal H3N2 virus, A/Oklahoma/309/06 (OK/06), and a prototypic H1N1 (PR8) strain in a human lung organ culture model. The influenza A(H1N1)pdm09 virus, A/Oklahoma/3052/09 (OK/09), is a clinical strain isolated during the 2009 H1N1 pandemic. After exposure of lung slice for 24 h to virus, infection was assessed by confocal immunofluorescence for intracellular viral nucleoprotein (NP). Replication was determined by quantitation of viral NP mRNA over time. Exposure of the tissue to OK/09 for 24 h resulted in detection of intracellular viral NP (red) associated with multiple cells, which appear morphologically to be alveolar epithelial cells and macrophages (Fig. 1A, b). Exposure of the tissue to PR8 for 24 h also resulted in detection of NP (red) (Fig. 1A, c). There was no detectable red fluorescence in tissue exposed to virus diluents, which contain the same buffer that viruses were diluted in, but contain no virus (Fig. 1A, a). Figures d-f of 1A are corresponding bright-field images which demonstrate that lung architecture is preserved during the experiment Thus, the human lung slices support infection during exposure to both influenza virus strains. To demonstrate the replication kinetics of these viruses, we measured NP mRNA expression in tissue slices by quantitative RT-PCR over 36 h of infection. The data demonstrated significant expression of viral NP mRNA upon virus inoculation (Fig. 1B). Assuming that the viruses infected relatively equal numbers of cells, initial replication rates of PR8 and OK/09 are quite similar and no significant difference was found except at 12 h after inoculation. In contrast, we found the H3N2 replicated faster than pandemic H1N1at 12, 24 and 36 h.

### Pandemic H1N1 Virus Induces a Diminished Antiviral Cytokine Response and RIG-I mRNA Expression in Human Lung as Compared with Prototypic Virus

**Figure 2:**
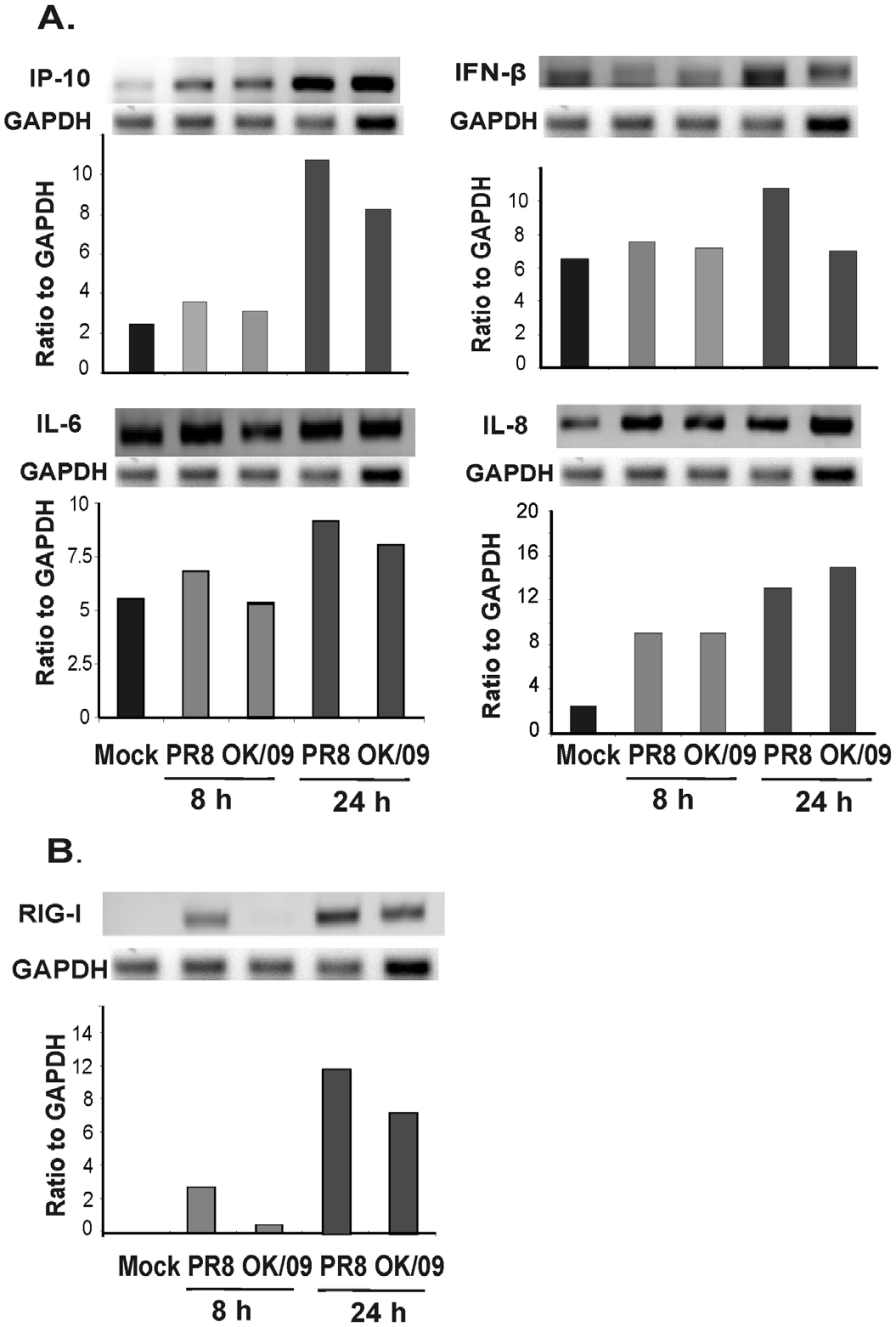
Pandemic OK/09 impairs host antiviral cytokine response and RIG-I response compared to PR8 in human lung. Human lung slices were exposed to 6×10^6^ PFU/ml of PR8 or OK/09 for 8 h and 24 h. Total RNA was then isolated from lung slices. Relative end-point RT-PCR products were separated by agarose gel electrophoresis, and mRNA expression was determined by densitometry of the appropriate bands on ethidium bromide-stained gels. Transcript levels of cytokines (*A*) and RIG-I (*B*) were normalized relative to the constitutively expressed GAPDH gene. Data are representative of 3 separate experiments.

We first examined the innate immune cytokine response to the pandemic and prototypic H1N1 strains in human lung. Lung slices were exposed to 6×10^6^ PFU/ml virus or virus diluent (negative control) for 8 and 24 h. Cytokines were measured in tissue slices by relative endpoint RT-PCR of mRNA levels. The mRNA induction patterns of IFN-β, interferon-inducible protein-10 (IP-10), interleukin (IL)-6 and IL-8 were substantially different between the two strains. Consistent with our earlier report, we found prototypic PR8 stimulates significant IP-10 and IFN-β induction [21], [30]. However, induction of IP-10 and IFN-β by OK/09 was substantially diminished compared to the PR8 stain. Specifically, IP-10 and IFN-β mRNA induction by OK/09 was less than that induced by PR8 at 24 h after infection (Fig. 2A). OK/09 virus induced a similar amount of the proinflammatory cytokine IL-6 as PR8 and higher IL-8 at 24 h although the induction by OK/09 is lower for both cytokines at 8 h. Our previous work and that of others implicates RIG-I in the induction of antiviral cytokine IP-10 and IFN-β, while proinflammatory cytokines IL-6 and IL-8 have been shown to be controlled by other pathways [21]. Therefore, we next sought to determine whether the diminished antiviral cytokine response by the pandemic stain could be due to decreased RIG-I induction by this virus (Fig. 2B). RIG-I expression induced by OK/09 was suppressed compared to PR8. These findings suggest that OK/09 causes immunosuppression of antiviral responses by escaping or suppression of the RIG-I mediated-sensing of viral infection.

**Figure 3:**
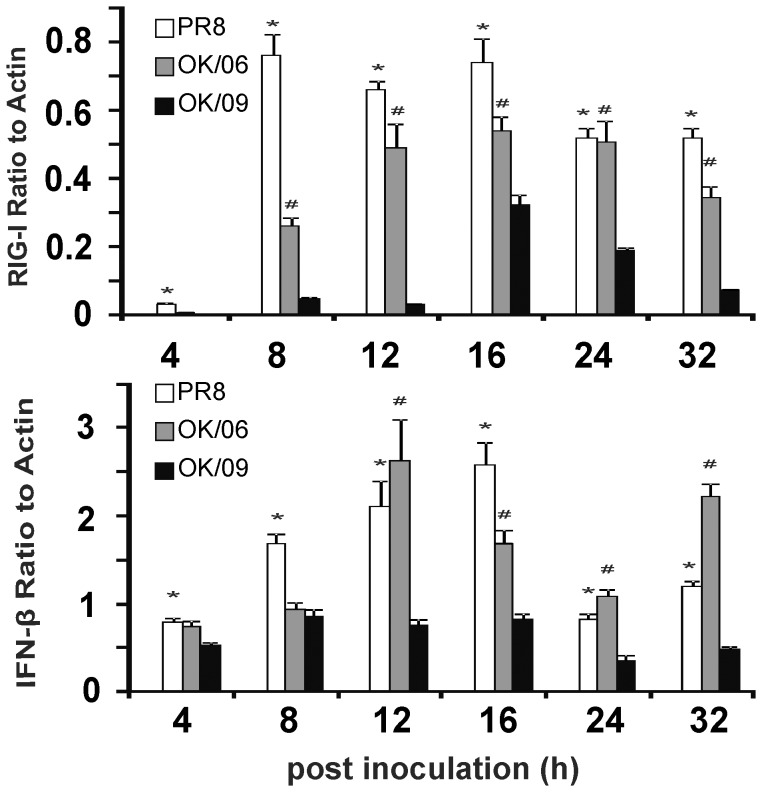
A time course study showing pandemic OK/09 impairs RIG-I and IFN-β antiviral immune responses compared to OK/06 and PR8 in human lung. Human lung slices were exposed to 6×10^6^ PFU/ml of PR8 or OK/09 for 4 h, 8 h, 12 h, 16 h, 24 h, and 32 h. Total RNA was then isolated from lung slices. Quantitative RT-PCR was performed using 100 ng sample RNA and SYBR Green (Quanta Biosciences) in a BIO-RAD iCycler IQ instrument (Hercules, CA). Transcript levels of RIG-1 (*A*) and IFN-β (*B*) were normalized relative to the constitutively expressed β-actin gene. Data are from 3 separate experiments. *, p<0.05 for PR8 to OK09. #, p<0.05 for OK/06 to OK/09.

To confirm differential viral immune responses to pandemic and seasonal influenza occurred, we examined the time course of mRNA induction of RIG-1 and IFN-β in human lung tissue to OK/09, OK/06 and PR8. Lung slices were exposed to 6×10^6^ PFU/ml virus or virus diluents (negative control) for 4, 8, 12, 16, 24 and 32 h and total RNA extracted for measurement of RIG-I and IFN- β levels by quantitative RT-PCR. Both viruses caused a time-dependent induction of RIG-1 and IFN-β in lung tissue. The peak induction of RIG-I by PR8 occurred between 8 h and 16 h post infection, while induction peaked at 16 h post infection for both OK/06 and OK/09. Importantly, RIG-1 expression was much lower in pandemic H1N1 OK/09 infected tissue than the other two viruses at all but the initial time point, 4 h. (Fig. 3A). The peak induction for IFN-β by all three stains occurred at 8–16 h post infection and IFN-β expression was much lower in lung infected with the pandemic strain than both of the other viruses at time points after 8 hours (Fig. 3B). Since PR8 and OK/09 have similar replication dynamics as we showed in Fig. 1B, differential induction is not attributable to different viral replication rates.

### Pandemic Virus Induction of RIG-1 and Antiviral Cytokine Protein in Human Lung is Reduced as Compared with Prototypic Virus

**Figure 4:**
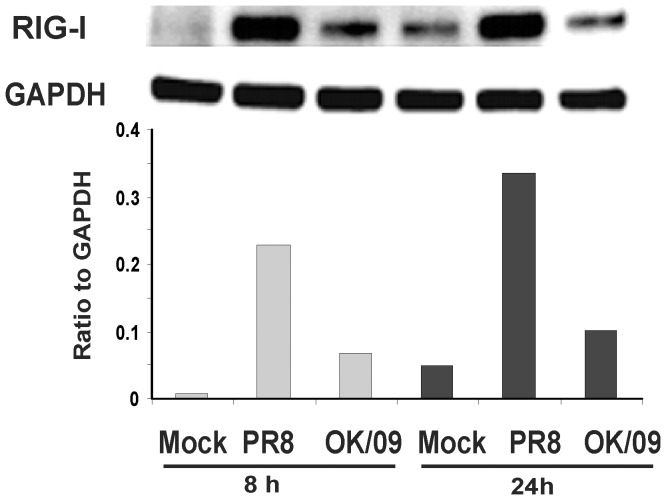
Pandemic OK/09 induces lower RIG-I protein responses than PR8 in human lung. Human lung slices were exposed to 6×10^6^ PFU/ml of PR8 or OK/09 for 8 h and 24 h. Western blot analysis was used to determine RIG-I protein expression in lung slices. Membranes were probed with anti-RIG-I or anti-GAPDH antibodies. Protein expression of RIG-I was normalized relative to GAPDH. Data are representative of 3 separate experiments.

RT-PCR data demonstrated that pandemic virus caused less induction of RIG-1 and anti-viral cytokine mRNA than seasonal virus, but similar induction of proinflammatory cytokine mRNA. To confirm that this differential mRNA expression was reflected at the level of translation, we measured RIG-1 protein in lung slices exposed to influenza virus at 8 and 24 h using Western Blot. We saw a significant induction of RIG-1 protein by PR8 virus while OK/09 induction of RIG-1 protein levels was only 30% of PR8 induction at 24 h (Fig. 4).

**Figure 5:**
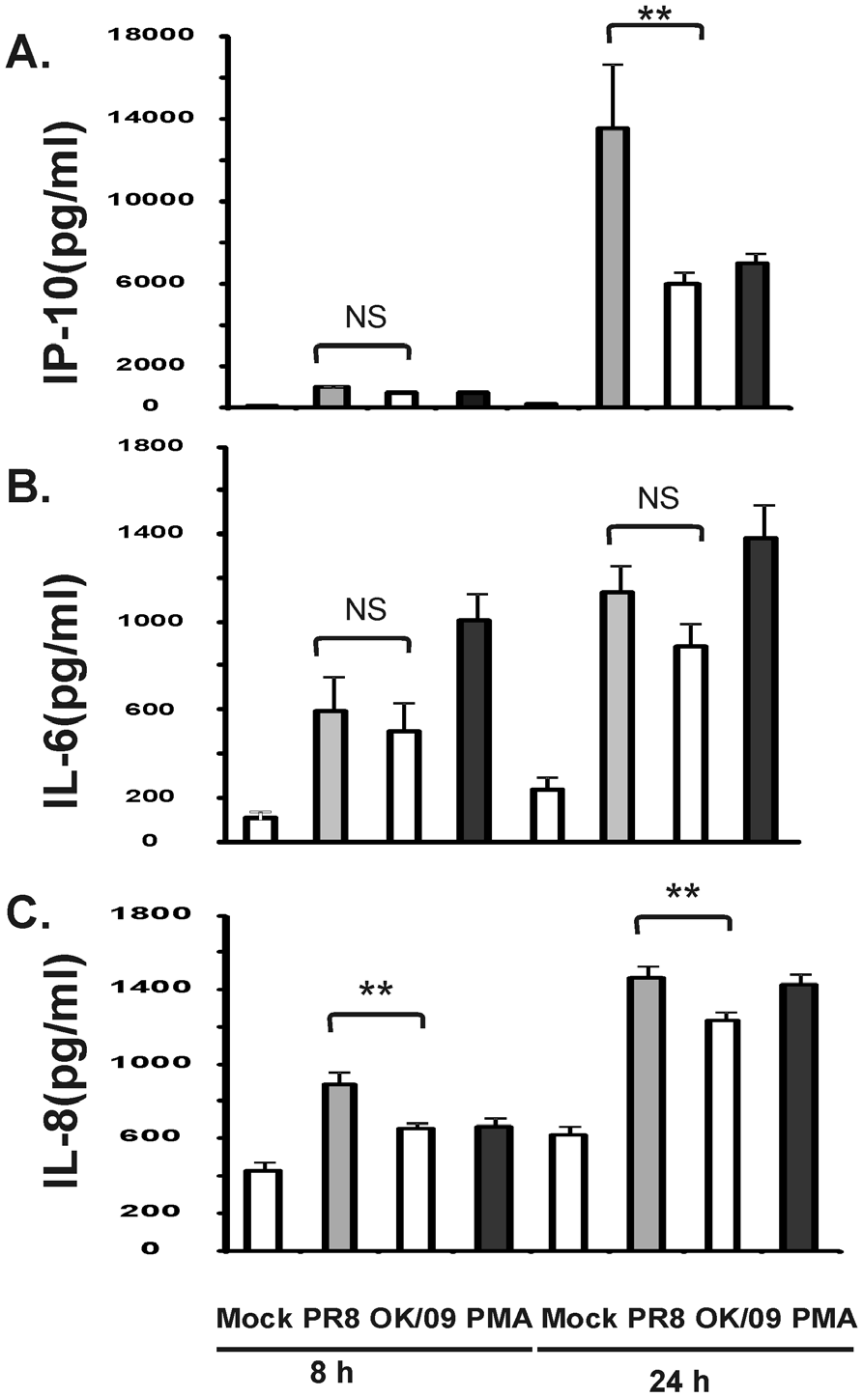
Pandemic OK/09 induces a diminished antiviral but not proinflammatory cytokine protein response as PR8 in human lung. For each data point, multiple lung slices were exposed to 6×10^6^ PFU/ml of influenza virus PR8 and OK/09 and allowed to incubate at 37°C, 5% CO_2_ for the indicated periods. Virus diluent was used as a negative control, and PMA (100 ng/ml) was used as a positive control. Chemokine and cytokine protein levels were determined by ELISA on lung slice supernatants. Data are expressed as the means ± SEM from three separate lung slice donor experiments. Statistical significance was determined by ANOVA. Means were compared to data from the negative control group.

We also tested for the corresponding cytokine proteins in the supernatants of lung slices exposed to influenza virus for 8 and 24 h using ELISA. Human lung slices were mock treated with virus diluents, 6×10^6^ PFU/ml of influenza virus, or treated with PMA (100 ng/ml) as a positive control. PR8 induced a 208–fold IP-10 and 3.4–fold IL-8 increase at 24 h after infection. In contrast, OK/09 induced a 91–fold IP-10 and 2.9–fold IL-8 increase at 24 h after infection respectively. This represents a 56% decline in IP-10 and a 16% decline IL-8 induction by OK/09 as compared with PR8 (P<0.05). There was no difference in the induction of IL-6 between the two viruses. Thus, consistent with the RT-PCR results, we found OK/09 caused a similar increase in the inflammatory cytokine IL-6, but lower anti-viral IP-10 protein induction compared to PR8 (Fig. 5). It should be noted that, although we saw a significant induction of IFN-β mRNA by PR8 virus, cytokine protein levels were below the limit of detection (100 pg/ml) by ELISA.

### Other Pattern Recognition Receptors in Pandemic Influenza Virus Infection in the Human Lung Model

**Figure 6:**
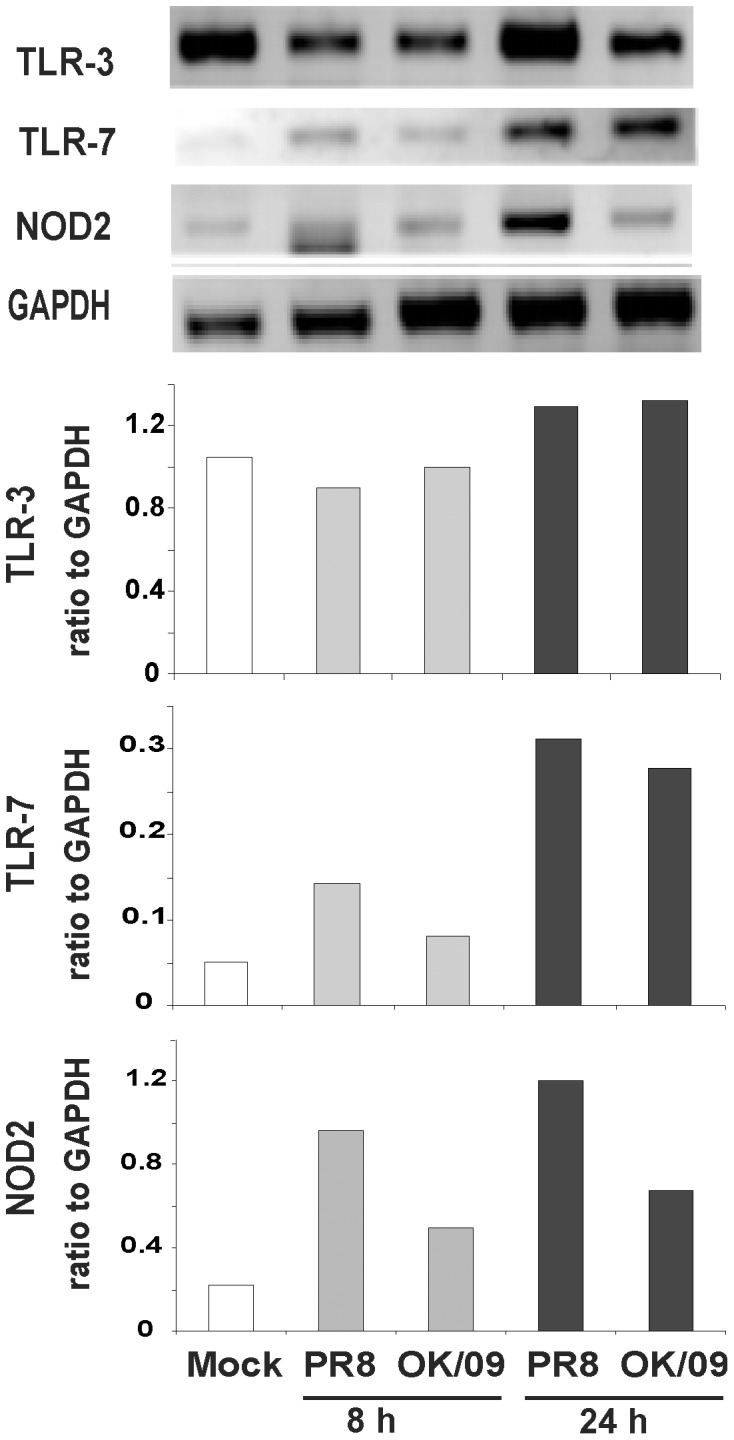
Pandemic OK/09 virus induces a similar TLR7 but diminished NOD2 mRNA expression compared to PR8 virus in human lung. Lung slices were exposed to 6×10^6^ PFU/ml of PR8 and OK/09 for 8 h and 24 h. Relative end-point RT-PCR was used to determine mRNA expression. Transcript levels of Toll-like receptor (TLR) 3, TLR 7, and nucleotide-binding oligomerization domain 2 (NOD2) were normalized relative to GAPDH mRNA expression. Data are representative of 3 separate experiments.

Influenza virus triggers intra- and extracellular receptors, called pattern recognition receptors (PRRs), during infection to elicit an innate immune response that serves as a first line of protection against infection. In addition to RIG-I [20], [23], influenza virus is also recognized by endosomal toll-like receptor (TLR) 3 and 7, and by Nucleotide-binding oligomerization domain-containing protein (NOD) 2 [31]. The influenza virus ssRNA genome is recognized by TLR7 in plasmacytoid dendritic cells (pDC) in humans [32]–[34]. Double-stranded RNA (dsRNA) is produced during viral replication and is recognized by endosomal TLR3 [35]. TLR3 is highly expressed in mouse innate immune cells, but shows a low level of expression in human monocytes, macrophages and dendritic cells [36]. We therefore investigated whether the other PRR’s were induced by influenza, and whether the responses were suppressed or differentially induced by the pandemic and seasonal influenza virus strains (Fig. 6). TLR3 was not significantly induced by virus compared to mock infection. TLR7 mRNA expression was significantly and equally induced by both PR8 and OK/09, about 6 fold over mock at 24 h after infection. In contrast, PR8 induced 5.5 fold increase of NOD2 while OK/09 only induced NOD2 by 3 fold. The data suggest that NOD2 induction may also be partially inhibited by OK/09.

### Cellular Source of RIG-I and IP-10 Induction by Influenza Virus

To determine which cell types in the lungs were responsible for RIG-I initiated antiviral cytokine response to the H1N1 strain, we performed immunohistochemistry on virus exposed lung slices. We examined RIG-I induction by influenza virus in epithelial cells and macrophages in the human lung. Lung slices were exposed to virus at 6×10^6^ PFU/ml or virus-free buffer for 24 h in the presence of brefeldin A to enhance the detection of cytokines. Slices were then processed for immunohistochemistry for the detection of influenza virus NP for viral infection, RIG-I and the cytokines IP-10. Macrophages and epithelial cells were detected using anti-CD68 monoclonal and anti-cytokeratin monoclonal antibodies, respectively. Tissues exposed to virus diluents were used to demonstrate basal cytokine detection. An additional negative control was performed for IP-10 and RIG-I detection using the same staining protocol but with IP-10 and RIG-I primary antibodies omitted. There was minimal background immunofluorescence in the absence of IP-10 and RIG-I primary antibodies (not shown).

**Figure 7:**
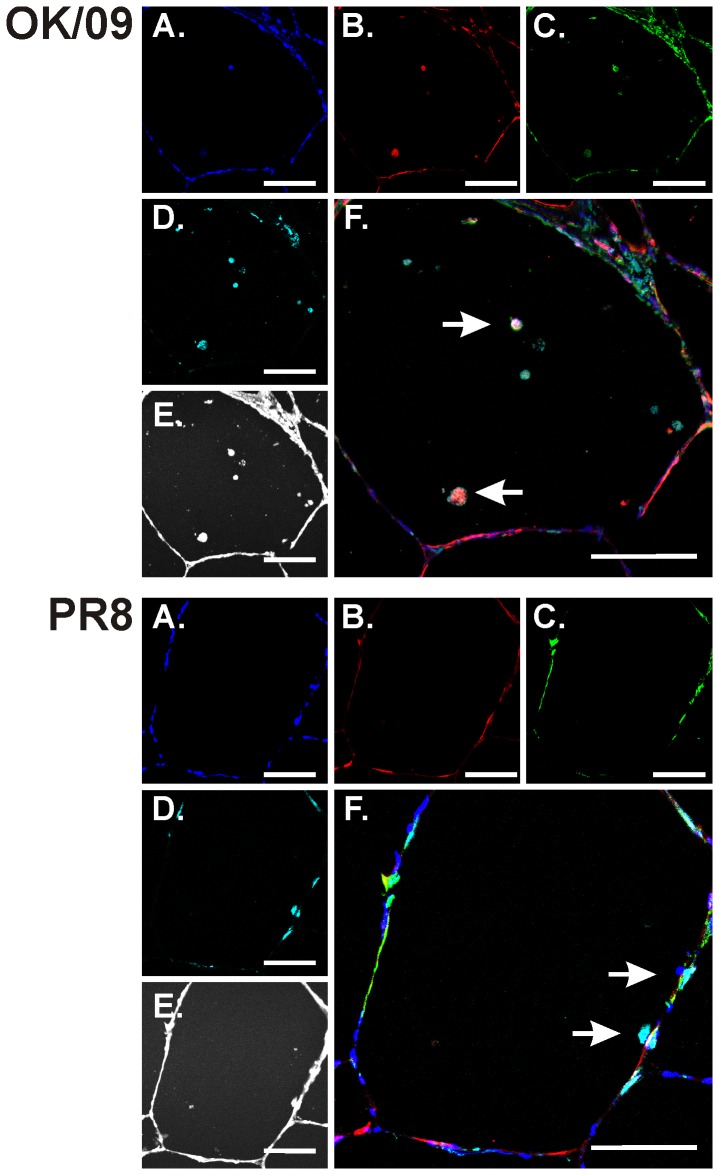
Infectivity of influenza virus and IP-10 induction in macrophages in human lung. Lung slices were exposed to 6×10^6^ PFU/ml of influenza virus PR8 or OK/09 or virus diluents for 24 h in the presence of brefeldin A (BFA) to enhance the detection of cytokines. Slices were then processed for immunohistochemistry for the detection of the chemokine IP-10 using goat polyclonal antibodies, viral nucleoprotein (NP) using rabbit polyclonal antibody, and macrophages using anti-CD68 monoclonal antibody. Nuclei were stained with SYTOX green. *Top*: OK/09. *Bottom*: PR8. *A–D*: fluorescent images that demonstrate nuclei (*A*; blue), NP (*B*; red), IP-10 (*C*; green), and macrophages (*D*; cyan). *E*: bright-field images that demonstrate that lung architecture is preserved during the experiment. *F*: overlays of the fluorescent images that demonstrate that influenza induces IP-10 in CD68 positive intraalveolar cells, likely alveolar macrophages (arrows). Bars = 100 µm.

**Figure 8:**
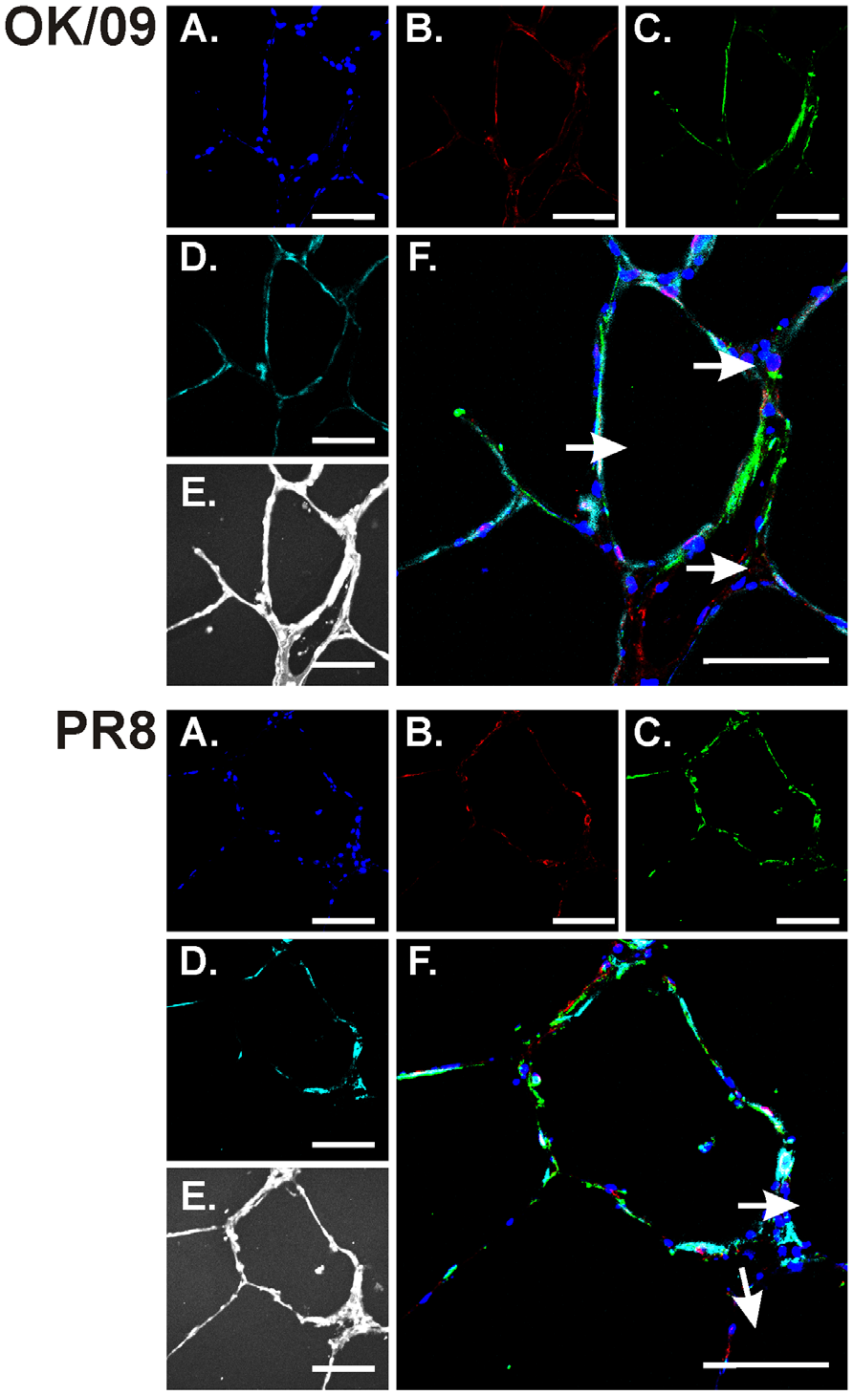
RIG-I and IP-10 induction in epithelial cells in the human lung. Lung slices were exposed to 6×10^6^ PFU/ml of influenza virus PR8 or OK/09 or virus diluents for 24 h in the presence of BFA to enhance the detection of cytokines. Slices were then processed for immunohistochemistry for the detection of IP-10 using goat polyclonal antibodies, RIG-I using rabbit polyclonal antibody, and epithelial cells using anti-cytokeratin monoclonal antibody. *Top*: OK/09. *Bottom*: PR8. *A–D*: fluorescent images that demonstrate nuclei (*A*; blue), RIG-I (*B*; red), IP-10 (*C*; green), and epithelial cells (*D*; cyan). *E*: bright-field images. *F*: overlays of the fluorescent images that demonstrate that influenza induces RIG-I in epithelial cells (arrows). Bars = 100 µm.

Detection of viral NP was observed after influenza virus infection (Fig. 7, B). OK/09 and PR8 both enhanced induction of IP-10 protein although IP-10 induction was less apparent in OK/09 infected cells (Fig. 7, C). RIG-I was also induced by both strains. Again, RIG-I induction was less prominent in OK/09 infected tissue (Fig. 8, B). In terms of cell types, OK/09 and PR8 infected both macrophages and epithelial cells of the human lung and IP-10 was produced by both types of cells (Figs. 7&8, C). There was significant overlap staining between RIG-I induction and epithelial cells, indicating that lung epithelia contribute to the innate immune response to influenza virus through RIG-I induction.

## Materials and Methods

### Preparation of Influenza Virus Stock

The viruses used in this study include a H1N1 influenza virus, A/PR/34/8 (PR8), a clinical isolate of the pandemic swine origin influenza A/H1N1 virus, A/Oklahoma/3052/09 (OK/09) and a clinical isolate of seasonal influenza A/H3N2 virus, A/Oklahoma/309/06 (OK/06). Both clinical isolates were isolated in the University of Oklahoma Health Science Center Clinical Microbiology laboratory. The viruses were passaged in Madin–Darby canine kidney (MDCK) cells. Viruses were grown in MDCK cells in DMEM/F12 with ITS+(BDBiosciences, Franklin Lakes, NJ ) and trypsin, harvested at 72 h postinfection and titered by plaque assay in MDCK cells. There was no detectable endotoxin in the final viral preparations used in the experiments as determined by limulus amebocyte lysate assay (Cambrex, Walkersville, MD). The lower limit of detection of this assay is 0.1 EU/ml or approximately 20 pg/ml LPS.

### Ethics Statement

Human lung tissue was obtained from patients undergoing resection for lung cancer in accordance with protocols approved by the Institutional Review Boards of the University of Oklahoma, Veterans Administration Hospital, Baptist-Integris Hospital, St. Anthony’s Hospital, and Mercy Health Center, all of Oklahoma City, OK (IRB #15358, Innate Response to Swine-Origin Influenza A/H1N1 Virus). Only tissue that did not contain tumor was used for experiments.

### Lung Explant Culture

The tumor-free lung tissue was transported in sterile PBS (pH 7.2) containing 200 µg of gentamicin/ml, 100 U of penicillin/ml, 100 µg of streptomycin/ml, and 2.5 µg of amphotericin B/ml (PBS+antibiotics) and the tissue was subsequently stored at 4°C in PBS+antibiotics for no longer than 4 h. The lung segments were inflated with lung slice medium (LSM) containing 1.5% agarose, 1 cm cores were prepared, and cores were sliced into 500 µm thick sections as described [29]. LSM consisted of minimal essential medium (Sigma, St.Louis, MO) supplemented with 1.0 µg of bovine insulin/ml, 0.1 µg of hydrocortisone/ml, 0.1 µg of retinyl acetate/ml, 200 µg of gentamicin/ml, 100 U of penicillin/ml, 100 µg of streptomycin/ml, and 1.25 µg of amphotericin B/ml. Each slice was placed in 0.5 ml of LSM in a single well of a 24-well plate, and then incubated at 37°C in 5% CO2. The LSM was replaced prior to subjecting the slices to the experimental treatments.

### Infection of Human Lung Slices with Influenza Virus and Determination of Cytokine Release by ELISA

After overnight incubation of the lung slices, the culture medium was replaced with fresh LSM. For each data point, three lung slices were each exposed to 6×10^6 ^PFU/ml of influenza virus OK/09 or PR8, and allowed to incubate at 37°C, 5% CO2 for the indicated periods. The amount of virus was derived from our previous publication, and represents about 6 MOI/cell (Wu et al. 2009). Virus diluent was used as a negative control, and PMA (100 ng/ml)/LPS (1 µg/ml) was used as a positive control. Following stimulation, media supernatants were harvested and stored at −20°C prior to ELISA. IP-10 were measured using commercially available ELISA kits (BD Biosciences, San Jose, CA). Finally, the plates were developed using the TMB reagent (BD Biosciences) and read using a Vmax kinetic microplate reader (Molecular Devices, Sunnyvale, CA) using an absorbance of 450 nm.

### RIG-I Protein Determination by Immunoblotting

Lung slices were stimulated with 6×10^6^ PFU/ml of influenza virus OK/09 or PR8 strain. Mock-infected, negative control slices were exposed to an equivalent volume of virus-free diluent. After 24 h incubation, the slices were harvested and homogenized, and then lysed in 500 µl of cold lysis buffer (150 mM NaCl; 50 mM Tris, pH 8.0; 10 mM EDTA, NaF, and sodium pyrophosphate; 1% NP-40; 0.5% sodium deoxycholate; 0.1% SDS; 10 µg of leupeptin/ml). Lung slice homogenates were clarified by centrifugation at 10,000 g, at 4°C for 10 minutes, and the clarified lysates were mixed with sodium dodecyl sulfate polyacrylamide gel electrophoresis (SDS-PAGE) sample buffer (60 mM Tris [pH 6.8], 10% glycerol, 2.3% SDS) and heated to 95°C for 5 minutes. The samples were separated by 4–15% gradient gel and electrophoretically transferred to Polyvinylidene Fluoride (PVDF) membranes. For the detection of proteins, the membranes were immunoblotted with rabbit polyclonal antibody specific for RIG-I (Abcam, Cambridge, MA) and GAPDH (R&D Systems). The membranes were developed with horseradish peroxidase-labeled goat anti-rabbit IgG (Cell Signaling Technology) and chemilluminescent reagents (Pierce Biotechnology, Rockford, IL).

Blots were developed using the Syngene G:box Bioimaging System and GeneTools software (Syngene, Frederick, MD) and quantified using ImageQuant software (BD/Molecular Dynamics, Bedford, MA).

### Measurement of mRNA Expression by RT-PCR

Total RNA from lung slices was extracted using a modified TRIzol (Invitrogen, Carlsbad, CA) protocol, spectrophometrically quantitated, and the integrity verified by formaldehyde agarose gel electrophoresis. Equal amounts (1 µg) of RNA for each sample were used with oligo (dT) as primers for production of cDNA (SuperScript II First-Strand Synthesis System for RT PCR, Invitrogen) to produce cDNA. Gene specific primers for the receptors and the actin housekeeping genes were used in standard PCR on a MJ Research DNA Engine thermal cycler with the following program: 1 cycle of 94°C for 2 min, followed by 32 cycles of 94°C for 30 sec, 56°C for 30 sec, 68°C for 2 min, and ending with a 68°C for 7 min extension. The primers’ sequences are as follow: RIG-I forward 5′- TCCTTTATGAGTATGTGGGCA-3′; RIG-I reverse 5′- TCGGGCACAGAATATCTTTG-3′; IFN-β forward 5′- GCTCTCCTGTTGTGCTTCTCCAC-3′; IFN-β reverse 5′-CAATAGTCTCATTCCAGCCAGTGC-3′; GAPDH forward 5′-GGAAGGTGAAGGTCGGAGT-3′; GAPDH reverse 5′- GAAGATGGTGATGGGATTTC-3′; TLR3 forward 5′-GTCTGGGAACATTTCTCTTC-3′; TLR3 reverse 5′-GCAGCTCTGCTGTTTCAGCAC-3′; NOD2 forward 5′-GAAGTACATCCGCACCGAG-3′; NOD2 reverse 5′-GACACCATCCATGAGAAGACAG-3′; IL-6 forward 5′-AGGAGCCCAGCTATGAACT-3′; IL-6 reverse 5′-TGAGATGCCGTCGAGGATG-3′;IL-8 forward 5′-GACTTCCAAGCTGGCCGTG-3′;IL-8 reverse 5′- 3′; IP-10 forward 5′-TCTAGAACCGTACGCTGTACCTGC-3′; IP-10 reverse 5′-209 CTGGTTTTAAGGAGATCT-3′; IFN- γ forward 5′-GGTCATTCAGATGTAGCGG-3′; IFN- γ reverse 5′-CACTCTCCTCTTTCCAATTC-3′. Following PCR, samples were separated on a 1.5% agarose gel, then stained with ethidium bromide (Invitrogen) for imaging and the band volumes were calculated using ImageQuant 5.0 software (Molecular Dynamics). Amplified DNA band densities were normalized to the corresponding actin densities to correct for potential differences in input cDNA. Quantitative RT-PCR was performed using 100 ng sample RNA and SYBR Green (Quanta Biosciences) in a BIO-RAD iCycler IQ instrument (Hercules, CA).

### Immunohistochemistry on Lung Tissue Explants

To examine which cell types were affected by influenza virus in the lung tissue, we performed immunohistochemical staining for IP-10, RIG-I and NP after influenza infection. Lung slices were exposed to 6×10^6^ PFU/ml of influenza virus or virus diluents. Brefeldin A (L C Laboratories, Wofford MA) was added at a concentration of 5 µg/ml to block protein export in order to enhance cytokine detection [29]. Following the incubation, the lung slices were fixed with 4% paraformaldehyde in PBS at room temperature for 30 minutes and were then imbedded in paraffin. Sections (3–5 µm) were mounted on glass slides and immuno-probed with a goat anti-human polyclonal antibody for IP-10 (R&D Systems), a rabbit anti-RIG-I polyclonal antibody (Abcam, Cambridge, MA), an anti-NP polyclonal antibody [37], an anti-CD 68 monoclonal antibody (Dakocytomation, Carpinteria CA) for macrophages or an anti-pan cytokeratin monoclonal antibody (Dakocytomation) for epithelial cells. After washing, the sections were probed with a donkey anti-goat secondary antibody conjugated to Alexa Fluor 350, a donkey anti-rabbit secondary antibody conjugated to Alexa Fluor 546 and a donkey anti-mouse secondary antibody conjugated to Alexa Fluor 647, and the cell nuclei were stained with SYTOX green (all from Molecular Probes). Transmitted light and fluorescent microscopy images were obtained using a Zeiss LSM-510 META Laser Scanning Confocal Microscope.

### Statistical Analysis

Where applicable, the data have been expressed as the means ± standard error of the mean (SEM). Statistical significance was determined by one-way ANOVA with Student-Newman-Keuls *post hoc* correction for multiple comparisons. Significance was considered as P<0.05.

## Discussion

The patterns of innate immune responses in patients with influenza A(H1N1)pdm09 virus infection have not been well characterized. The key puzzle is whether severe illness is the result of enhanced virus replication, broader cellular receptor binding, viral mediated immunosuppression of the host, or a massive cytokine storm. In the present study, we compared a influenza A(H1N1)pdm09 virus, a seasonal H3N2 strain and a prototypic H1N1 strain, and examined whether immunosuppression is involved in the innate immune responses to pandemic H1N1 virus in a human lung organ culture model.

A study using carbohydrate microarray showed that the pandemic virus binds to α2-3- in addition to α2-6-linked sialic acid receptors, which might be pertinent to the greater virulence of the pandemic virus than seasonal influenza viruses observed in animal models [38], and its capacity to cause severe and fatal disease in humans, despite the generally mild nature of most infections [39]. Influenza affects both the upper and lower respiratory tracts. Lower respiratory tract infections of influenza are generally more serious than upper respiratory infections and can cause severe viral pneumonia. The α2-6-linked sialyl glycans predominate in the upper respiratory tracts while the α2-3 is most expressed in the lower respiratory tracts such as alveoli, bronchia and other lung cells. Binding to α2-3-linked sialic acid is thought to be associated with the ability of influenza virus to infect the lower respiratory tract. The α2-3-linked receptor binding ability of the influenza A(H1N1)pdm09 virus was confirmed by another group using the human conjunctival epithelium, which has been reported to lack α2-6-linked receptor but expresses the α2-3-linked receptor [40]. Of note, there are limitations of using carborhydrate microarrays to determine binding sites. They do not cover every single sialyated glycan that present in human respiratory tract. Seasonal H1N1 can also bind both α2-3- and α2-6- linked glycans [41]. In addition, whether or not binding sites differ, similar replication kinetics between pdm09 and seasonal virus are present in lower lung [40]. Therefore, the intensity of virus infection does not explain why more pandemic flu patients experience severe low respiratory tract symptoms.

Pathological evaluation of respiratory specimens from initial 2009 influenza-associated deaths revealed that the most prominent histopathological feature observed was diffuse alveolar damage in the lung in all patients examined. Alveolar lining cells, including type I and type II pneumocytes, were the primary cells infected [42]. Our model is thus relevant in this context as it consists of distal parenchymal lung tissue, the site of pandemic virus infection. Our immunohistochemistry results showed that both viruses infected alveolar macrophages and epithelial cells. The findings regarding the pandemic virus is consisted with other work showing that pandemic virus can infect lung DC and AM [43]. The findings regarding seasonal influenza are consistent with our work showing seasonal H1N1 PR8 and H3N2 virus infected and replicated in our human lung model [30]. Our current and previous findings showing that both pandemic and seasonal influenza infect the lower respiratory tract suggest that there are additional reasons besides differential receptor binding affinity for the increased morbidity and mortality during pandemic influenza infection.

One explanation is our major finding that influenza A(H1N1)pdm09 virus, relative to prototypic H1N1 influenza, suppresses viral-mediated induction of RIG-I and the RIG-I initiated anti-viral immune responses in infected human lung. Pandemic virus partially inhibited RIG-I expression, and the subsequent antiviral cytokine IP-10 and IFN-β response in human lung. However, induction of the proinflammatory cytokines, IL-8 and IL-6, at both the transcriptional and translational level are similar between the pandemic virus OK/09 and PR8. The differences in antiviral cytokine induction in our model is consistent with clinical findings that reported different cytokine response patterns in adults hospitalized for severe pandemic H1N1 and seasonal influenza [44]. In patients with pandemic H1N1 pneumonia, the adaptive Th1/Th17-immunity related cytokines (e.g. IP-10, MIG, IL-17A) were markedly suppressed. Österlund et al also demonstrated that the pandemic 2009 H1N1 virus induced a diminished antiviral response, as evidenced by a diminished induction of IFN-α, IFN-β, IFN-λ1, and IFN-λ2/3 genes in primary human macrophages and DCs [43].

Sensing of virus presence and cytokine induction via the RIG-I pathway are crucial for successful host defense against infections with RNA viruses [24]. We demonstrate here that antiviral cytokine suppression is caused by pandemic virus-mediated inhibition of RIG-I induction. During influenza infection, an effective immune response needs well-regulated integration between innate and adaptive immunity. IFN-α and IFN-β inhibit the replication of the pandemic H1N1 virus [43]. IP-10 and its stimulator, IFN-γ, coordinately regulate the Th1 cellular immune response, also important for virus control during infection [45]. However, induction of inflammatory cytokines, which are not triggered by RIG-I, were at the same level by pandemic and seasonal virus. In fact, increased plasma levels of IL-15, IL-12p70, IL-8, and especially IL-6 may be markers of critical illness [46]. Thus, the defect in immune response is not due to inflammatory failure, but is due to the failure of the induction of antiviral cytokine and RIG-I suppression while proinflammatory cytokine induction is unimpaired. Our findings suggest that RIG-I related cytokine dysfunction may play an important role in the disease pathogenesis by facilitating escape of pandemic virus from the innate immune antiviral cytokine responses.

RIG-I induction may also be caused in a paracrine fashion. Hui et al investigated the effect of influenza virus infection and infected culture supernatants on the expression of PRRs [47]. The mRNA expression of RIG-I, MDA5, and TLR3 was markedly upregulated directly by viral infection and also by treatment of uninfected cells with virus-free supernatant. Upregulation of RIG-I of uninfected cells further enhanced the cytokine expression to virus infection. The findings demonstrated here do not distinguish between a direct or indirect effect of virus infection on RIG-I. It is possible that pandemic virus might act by preventing infected cells from inducing RIG-I in neighboring uninfected cells.

There are two other families of PRRs besides the RIG-I like helicases: the TLRs, and the nucleotide-binding domain and leucine-rich-repeat-containing proteins (NLRs). All three families are involved in recognition of influenza virus, and studies in cell culture and mouse models show that they cooperatively operate to coordinate the response to the virus [48]. We examined whether TLR or NLR suppression could play a role in the increased pathogenesis of pandemic influenza infection. We found, consisted with findings in isolated cells by other investigators that TLR7 is induced by influenza virus in human lung [32], [33]. However, we found that TLR7 was not suppressed by pandemic influenza, suggesting it does not play a role in the increased pathogenesis of this strain. TLR3 was not significantly induced by both influenza strains. We also measured regulation of one of the NLR, NOD2, in our model. This is a cytoplasmic protein that detects bacterial molecules which possess the muramyl dipeptide (MDP) moiety and activates the NF-κB protein. It also facilitates activation of IRF3 and production of IFN-β induced by ssRNA and by RNA viruses, including influenza virus [31]. In our model, we found NOD2 induction was also inhibited by pandemic influenza virus. Interestingly, that RIG-I and NOD2 form a direct interaction at actin enriched sites within cells, and RIG-I negatively regulates ligand-induced nuclear factor-κB (NF-κB) signaling mediated by NOD2. NOD2 also negatively regulates type I interferon induction by RIG-I [49]. Further study will be necessary to whether NOD2 immunosuppression by pandemic influenza is related to its interaction with RIG-I, or is an independent phenomena.
